# Perioperative FOLFOX in management of peritoneal metastases of colorectal cancer. Case report of 2 patients

**DOI:** 10.1016/j.ijscr.2020.09.017

**Published:** 2020-09-10

**Authors:** Paul H. Sugarbaker, O. Anthony Stuart

**Affiliations:** Program in Peritoneal Surface Malignancies, MedStar Washington Hospital Center, Washington, DC, USA

**Keywords:** Peritoneal metastases, Cytoreductive surgery, Peritonectomy, 5-fluorouracil, Oxaliplatin, Leucovorin, Regional chemotherapy, FOLFOX, Colorectal cancer, Cytology, Adjuvant treatment, HIPEC, CHIP, Case report

## Abstract

**Introduction:**

Colorectal cancer can disseminate malignant cells to the peritoneal surfaces which over time progress to peritoneal metastases. Management of this type of metastatic disease has been approached using a combined treatment that consists of cytoreductive surgery and perioperative chemotherapy. To optimize these treatments a more effective chemotherapy that is used as planned part of the surgical procedure is required.

**Presentation of cases:**

Pharmacologic studies to initiate a new perioperative chemotherapy treatment were modeled after the successful systemic treatments of metastatic colorectal cancer referred to as FOLFOX. A management plan that included all of the essential features of successful systemic chemotherapy was formulated. Pharmacokinetic studies of 5-fluorouracil given both intravenously and intraperitoneally and oxaliplatin given intraperitoneally were investigated.

**Discussion:**

The compatibility of 5-fluorouracil and oxaliplatin was documented showing no degradation of either drug when they were mixed *in-vitro* over 24 h. The pharmacokinetic analysis of hyperthermic intraperitoneal chemotherapy (HIPEC) with oxaliplatin showed cytotoxic concentrations for 120 min. In order to maximize oxaliplatin’s effect, both intravenous bolus and continuous infusion 5-fluorouracil was combined with early postoperative intraperitoneal chemotherapy (EPIC). In total, 200 mg/m^2^ of oxaliplatin was combined with a minimum of 1600 mg of 5-fluorouracil over the 24 -h treatment plan.

**Conclusion:**

This perioperative FOLFOX treatment was completed in 2 patients and the clinical effectiveness as a result of this in-depth case reports was presented. Formal phase II studies, as a result of these pharmacokinetic and clinical investigations, have been initiated.

## Highlights

• Peritoneal metastases from colorectal cancer are lethal.
• Cytoreductive surgery plus perioperative chemotherapy is a treatment option.
• A perioperative FOLFOX regimen with intraperitoneal oxaliplatin was described.
• The pharmacokinetic studies suggested efficacy.

## 1 Introduction

Peritoneal metastases from gastrointestinal cancer has been regarded as a lethal condition with limited survival following confirmed diagnosis [[1], [2], [3]]. In the early 1990s, reports of peritoneal metastases prevention using perioperative intraperitoneal chemotherapy appeared. The pharmacologic rationale [4] was published and a randomized controlled trial supporting the efficacy of intraperitoneal chemotherapy to prevent peritoneal metastases were published [5]. Manuscripts to support treatment of peritoneal metastases from appendiceal and colorectal cancer were published in 1995 [6]. The treatment required a combination of peritonectomy procedures and visceral resections to remove all visible evidence of disease. Following the surgery, the abdomen and pelvis was flooded with a large volume of intraperitoneal chemotherapy solution to eradicate microscopic residual disease. Although the surgical part of this combined treatment has been standardized, there are many different perioperative chemotherapy regimens for colorectal peritoneal metastases currently in use. No consensus has been reached.

Further confusion regarding optimal perioperative chemotherapy treatments was created by the negative PRODIGE 7 randomized controlled trial reported by the French peritoneal surface oncology group at American Society of Clinical Oncology Meeting in 2018 [7]. Using chemo-hyperthermia intraperitoneal (CHIP) after complete cytoreduction of colorectal peritoneal metastases, no benefit occurred in a randomized controlled trial of 265 patients. The CHIP perioperative chemotherapy regimen used by this French group has been criticized for inadequate augmentation of oxaliplatin by 5-fluorouracil, brief direct contact of chemotherapy with tissue, and CHIP used after maximal systemic oxaliplatin treatment in 84 % of patients.

In this report of two patients treated for appendiceal adenocarcinoma with peritoneal metastases, the use of a prolonged hyperthermic perioperative chemotherapy (HIPEC) with oxaliplatin simultaneously with adequate doses of 5-fluorouracil to synergize oxaliplatin is described. A clinical and pharmacologic rationale for perioperative oxaliplatin plus 5-fluorouracil was presented and shown to be theoretically far superior to the CHIP regimen reported in the French randomized controlled trial. A controlled trial with perioperative FOLFOX is indicated.

Data on our 2 patients was prospectively recorded and then retrospectively reviewed at an academic institution. This research work has been reported in line with the SCARE criteria [8]. This study was registered as a case report on the www.researchregistry.com website with UIN 5425. Pharmacologic monitoring is a routine part of perioperative chemotherapy administration at this institution and is part of an ongoing quality improvement project [9]. Written informed consent was obtained from our two patients for publication of these case reports. A copy of the written consent is available for review by the Editor-in-Chief of this journal upon request.

## 2 Case presentations

### 2.1 Overview of treatments administered

Both patients had an invasive appendiceal cancer with peritoneal metastases appropriate for combined treatment with cytoreductive surgery and perioperative chemotherapy. Permission to publish these clinical and pharmacologic information was obtained in writing from our two patients. They were adequately consented for all treatments. Cytoreductive surgery was performed prior to any perioperative cancer chemotherapy in order to reduce the volume of cancer to a microscopic amount [10]. All surgical procedures were performed by PHS. Perioperative chemotherapy was given intravenously and intraperitoneally in the operating room and in the early postoperative period [11]. All chemotherapy administration was supervised by PHS. In the operating room during the HIPEC procedure, aliquots to chemotherapy solution were taken directly from the chemotherapy solution in the peritoneal space and from the blood.

### 2.2 Pharmacologic monitoring of intraperitoneal and intravenous chemotherapy

High performance liquid chromatography (HPLC) assay for oxaliplatin was performed by OAS, a biochemist with over 30 years’ experience. The pharmacologic monitoring was similar to that by Mehta et al. [12]. Our method was with UV detection using an 1110 series HPLC system consisting of a binary pump, Model G1312A, an autosampler Model G1367A and a UV-detector Model G1314A (Agilent Technologies, Santa Clara, CA, USA). A Hypersil ODS analytical column (250 × 4.6 mm ID, particle size 5 μm, Thermo Scientific, Waltham, WA, USA) was used. Absorbance was measured at 210 nm. Injection of 20 μl of sample was followed by isocratic elution with 10 % of acetonitrile (Biosolve B.V., Amsterdam, The Netherlands) in water for injections (B. Braun, Melsungen, Germany) set at pH 3.0 using ortho-phosphoric acid (Merck, Darmstadt, Germany). The flow rate was 1.2 ml/min, with a total runtime of 12 min. Chromatograms were processed using Chromeleon software (Thermo Scientific).

For 5-fluorouracil, the HPLC assays were performed as described by Jacquet et al. [13]. Five hundred μl of sample (serum, plasma, or peritoneal fluid), 25 μl of internal standard (50 μM 5-chlorouracil in water), and 50 μl 1.0 M potassium phosphate buffer, pH 7.0, was added to a tube and vortexed. The drug was extracted with 8 ml ethyl acetate; the organic phase was recovered and evaporated to dryness. Samples were reconstituted with 150 ul of the mobile phase. Mobile phase constituted of 20 mM acetic acid in 1 % acetonitrile. Instrumentation included a 510 HPLC pump, a 710-B WISP auto sampler, a RCM100 Radial Compression Module containing a Radial-Pak C_18_-μBondapak column, a model 481 UV detector, (all from Waters, Inc., Milford, MD, USA), and a C-R6A integrator/recorder (Shimadzu Instruments, Columbia, MD, USA). The flow rate was 1.0 ml/min and the detector was set at 266 nm and 0.001 ultraviolet absorbance (AUFS). Late eluding peaks were flushed from the system by injection of 300 μl of acetonitrile between each analytical run. The HPLC system consisted of a Shimadzu LC7A instrument equipped with an SPD-6AV (UV–vis) detector set at 295 nm and a C-R8a ‘Chromatopac’ data processor (Shimadzu Instruments, Columbia, MD, USA). A reversed C18 column (Varian Associates, Walnut Creek, CA, USA) was used for chromatographic separation. The mobile phase consisted of 28 % acetonitrile in 0.1 % orthophosphoric acid with 0.1 % triethylamine. The flow rate was 1.2 ml/min and samples were injected through a 50 μl injector loop.

### 2.3 *In-vitro* studies to test the molecular integrity of oxaliplatin, 5-fluorouracil, and an oxaliplatin plus 5-fluorouracil mixture

In this study all of the chemotherapy tested *in-vitro* was diluted in a 1.5 % dextrose peritoneal dialysis solution (Dianeal, Baxter Healthcare, Deerfield, IL). This is the same carrier solution for intraperitoneal chemotherapy used in patients. High pressure liquid chromatography (HPLC) was used to monitor the molecular stability of both 5-fluorouracil and oxaliplatin [12,13]. The molecular integrity was determined for each drug individually and with both drugs mixed together. A low and high concentration of drugs were similar to those used in the intraperitoneal chemotherapy for a patient with body surface area of 2.0 m^2^. The *in-vitro* tests were performed over 24 h. Because the intraperitoneal chemotherapy was used at moderate heat, the stability of these drugs as assessed by HPLC was determined at room temperature and at 37 °C. Samples were assayed at 0, 1, 2, 4, 6 and 24 h.

### 2.4 Components of the perioperative FOLFOX regimen

**Fig. 1:**
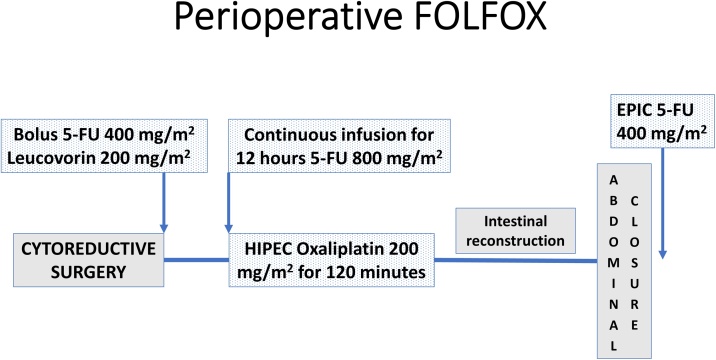
Diagram illustrating the intravenous and intraperitoneal administration of 5-fluorouracil and leucovorin to synergize the intraperitoneal oxaliplatin cytotoxicity.

Oxaliplatin was instilled at 1.5 L/m^2^ of peritoneal dialysis solution at 200 mg/m^2^ at the beginning of the hyperthermic intraperitoneal chemotherapy (HIPEC) using the open technique [14]. The intraperitoneal lavage was continued for 120 min. At appropriate intervals, to achieve a pharmacokinetic analysis, 2 ml aliquots of chemotherapy solution were extracted from the peritoneal space. Intravenous analysis of oxaliplatin concentrations was not performed. A diagram to clarify the intravenous and intraperitoneal chemotherapy administration is presented as Fig. 1.

### 2.5 Intravenous 5-fluorouracil and leucovorin administration at the initiation of intraperitoneal chemotherapy

Fifteen minutes prior to the initiation of intraperitoneal administration of oxaliplatin, a bolus intravenous infusion through a peripheral vein of 5-fluorouracil at 400 mg/m^2^ was begun. A separate intravenous infusion of leucovorin at 200 mg/m^2^ through a separate peripheral vein was simultaneously initiated. These two infusions required approximately 8 min.

### 2.6 Continuous infusion of 5-fluorouracil for 12 h

As soon as the 120-minute HIPEC with oxaliplatin is begun, a continuous infusion 800 mg/m^2^ of 5-fluorouracil is begun. The drug diluted in 250 ml normal saline was administered over the next 12 h.

### 2.7 Early postoperative intraperitoneal chemotherapy (EPIC) with 5-fluorouracil administered in the operating room

After the abdomen was closed and the drains positioned, a single Tenckhoff catheter was positioned through the lateral aspect of the abdominal wall. In order for this instillation to be maximally effective the midline fascia of the abdominal wall incision must be closed in a watertight manner. Also, purse string sutures are placed around all drains that pass through the abdominal wall and the drains are clamped prior to the intraperitoneal infusion. The 5-fluorouracil is at 400 mg/m^2^ in 1 L of 1.5 % dextrose peritoneal dialysis solution. It is infused by gravity into the peritoneal space. The chemotherapy solution is allowed to dwell for 24 h. At 24 h postoperatively, all transabdominal drains are opened to empty the abdomen as completely as possible of serosanguineous fluid and chemotherapy solution. In patients with little or no bone marrow suppression from prior systemic chemotherapy, additional daily instillations of EPIC 5-fluorouracil can be given at the discretion of the surgical oncologist.

## 3 Patient presentations

### 3.1 Patient 1

A 37-year-old woman presented to the Washington Cancer Institute with a recurrence of mucinous adenocarcinoma of the appendix. There was no family history of appendiceal or colorectal cancer. This was thought to be a sporadic and not familial cancer. CEA blood test had increase from normal to 7 mg/mL and 3 lesions were detected by CT scan within the abdomen.

Prior to the current intervention the patient had two prior cytoreductions with perioperative chemotherapy. The first was on 05/13/2009. After an 11½-hour cytoreduction she was treated with HIPEC mitomycin C and EPIC 5-fluorouracil. The second operation was on 06/05/2012. After this 7 -h operation she was treated with hyperthermic intraperitoneal melphalan.

On 12/20/2018, she underwent a third cytoreductive surgery for 11 h. All visible tumor was removed. The HIPEC was oxaliplatin at 200 mg/m^2^. She also received 400 mg of intravenous 5-fluorouracil by bolus and 800 mg/m^2^ by continuous infusion following the HIPEC. Postoperatively, EPIC treatment was with 5-fluorouracil at 400 mg/m^2^ for two instillations.

The patient’s postoperative course was benign until postoperative day 13 when she had a CT scan which showed approximately 1 L of intraperitoneal blood. She was taken back to the operating room and this was evacuated without incident. She was discharged from the hospital on her 19th postoperative day with minimal discomfort and eating without intravenous supplementation. Currently, 20 months postoperatively she has no evidence of disease. No further cancer chemotherapy treatments are planned unless recurrent disease is documented by serial CEA blood tests and CT follow-up.

### 3.2 Patient 2

A 59-year-old woman presented to the Washington Cancer Institute with a recurrence of intestinal-type (non-mucinous) appendiceal adenocarcinoma. There was no family history of appendiceal or colorectal cancer. This was thought to be a sporadic and not familial cancer. Her visit was prompted by a mass in the right iliac fossa on PET-CT and a rapidly increasing CA 19-9. She was asymptomatic.

On 02/02/2017, right lower quadrant pain caused a diagnosis of appendicitis. At surgery an adenocarcinoma of the base of the appendix was treated by ileocaecectomy. Two lymph nodes were negative for cancer.

On 10/12/2017, recurrent disease at the ileocaecectomy site was resected.

On 03/17/2018, a third intervention was a sigmoid colon resection for limited peritoneal metastases.

On 05/04/2019, PET-CT showed a mass in the right iliac fossa and a rising CA 19-9 to 114 units/mL. Upper and lower gastrointestinal endoscopy were normal.

On 06/19/2019, a cytoreductive surgery was performed. Tumor was resected from the right paracaval and right common iliac lymph node chain. Cancer dissemination beneath the bifurcation of the common iliac artery and vein and going down the femoral canal into the upper thigh was not resected. There was tumor spillage with the extensive lymphadenectomy.

The hyperthermic perioperative chemotherapy was FOLFOX and a single instillation of EPIC 5-fluorouracil. Following perioperative FOLFOX areas of residual cancer were marked out by metal clips for possible postoperative radiotherapy. Sites of residual disease were excluded from the abdomen and pelvis by an omental pedicle flap. There were no postoperative complications. Function of the right lower extremity returned to normal. At 10 months after cytoreductive surgery, no disease progression has occurred with the patient on systemic chemotherapy. Monitoring for possible disease progression is by serial CA 19-9 blood tests and follow-up CT.

## 4 Results

### 4.1 *In-vitro* HPLC analysis of oxaliplatin and 5-fluorouracil

When 5-fluorouracil was incubated at room temperature or 37 °C for 24 h, no changes in the molecular structure of the drug as seen by HPLC analysis occurred. All HPLC spikes and concentrations remained intact with no degradation of the molecule detected. The concentration of oxaliplatin remained unchanged for 6 h, both at room temperature and at 37 °C. However, at 24 h there was a deterioration of the drug (approximately 20 % at room temperature and 30 % at 37 °C. When the mixture of oxaliplatin and 5-fluorouracil was analyzed no evidence of drug interaction (complexes) as assessed by HPLC was detected for 24 h at room temperature or 37 °C. Our conclusion was that no drug complexes of oxaliplatin and 5-fluorouracil formed. If these two drugs did not complex with each other over 24 h of *in-vitro* surveillance, drug interactions within the peritoneal space over similar temperatures and in the same carrier solution are unlikely to occur.

### 4.2 Intraperitoneal chemotherapy administered oxaliplatin

Oxaliplatin at 200 mg/m^2^ was diluted in 2 L of 1.5 % dextrose peritoneal dialysis solution. The instillation was as rapid as possible through two Tenckhoff catheters using a hyperthermia pump (Belmont Medical Technologies, Billerica, MA). The temperature of the solution as it was being infused was 44 °C and the temperature in the abdomen and pelvis was 41 °C. The rate of infusion was 700 ml/minute. The open method of chemotherapy administration with manual distribution was used to facilitate the distribution of the oxaliplatin solution (14).

**Fig. 2:**
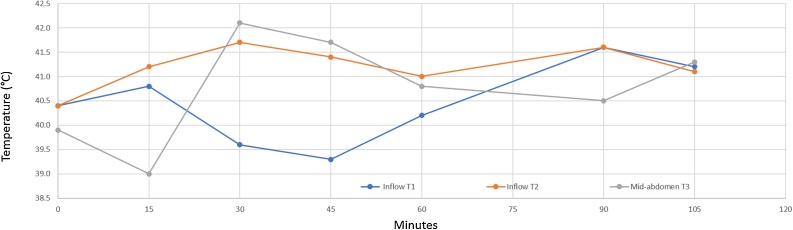
Temperature at two inflow sites and a mid-abdomen site when hyperthermic intraperitoneal chemotherapy was administered by the open method for 120 min.

The heat in the peritoneal space was monitored at three sites. The temperature probes were positioned at the inflow beneath the diaphragm, at the inflow within the pelvis, and within the mid-abdomen. Continuous manual distribution of the chemotherapy was to maintain approximately the same 41 °C temperature in all parts of the abdomen. Fig. 2 shows the temperatures maintained during the HIPEC with oxaliplatin for 120 min in patient 2.

The pharmacokinetics of intravenous and intraperitoneal 5-fluorouracil and intraperitoneal oxaliplatin were monitored in the two patients presented.

### 4.3 Pharmacokinetics of HIPEC oxaliplatin

**Fig. 3:**
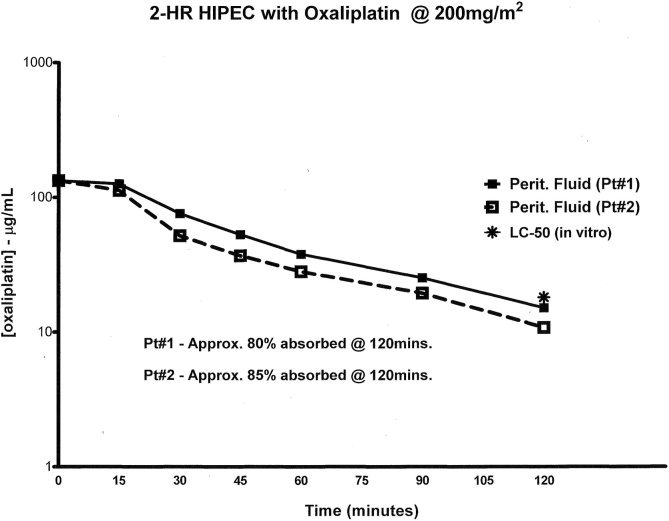
Pharmacokinetic analysis of intraperitoneal oxaliplatin determined over 120 min in two patients. Lethal concentration of *in-vitro* of oxaliplatin for 50 % colon cancer cells untreated by neoadjuvant chemotherapy was 18.1 μg/mL.

The intraperitoneal concentration of oxaliplatin over the 120 min of HIPEC for patients 1 and 2 is shown in Fig. 3. At the end of HIPEC 90 % oxaliplatin had cleared from the peritoneal space in patient 1 and 85 % in patient 2. Concentration of oxaliplatin in the chemotherapy solution after 120 min in patient 1 and 2 was 15.15 μg/mL and 10.81 μg/mL, respectively.

### 4.4 Pharmacokinetics of intravenous 5-fluorouracil by bolus and continuous infusion during perioperative FOLFOX chemotherapy

**Fig. 4:**
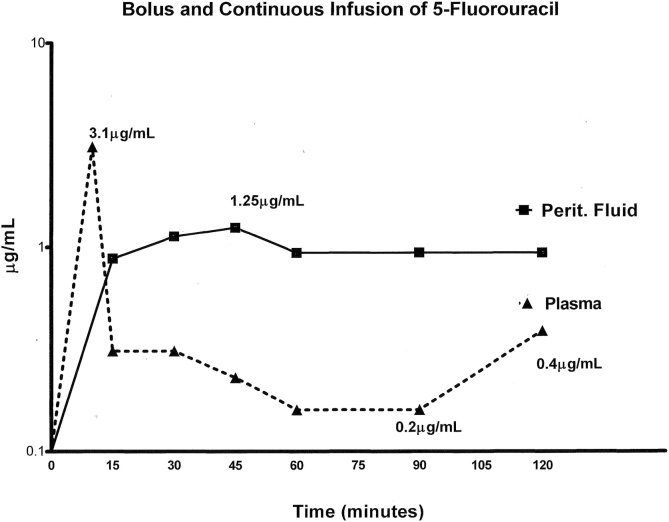
Concentration over time for 5-fluorouracil. Bolus intravenous administration of 400 mg/m^2^ of 5-fluorouracil occurred 15 min prior to the initiation of HIPEC. At the same time HIPEC oxaliplatin was started, 800 mg continuous intravenous infusion of 5-fluorouracil over 12 h was begun.

The intravenous bolus and then continuous infusion of 5-fluorouracil concentrations are shown in Fig. 4. The bolus intravenous infusion causes a transient spike in the plasma 5-fluorouracil. Then as expected from prior pharmacokinetic studies, the intraperitoneal concentration of drug exceeds the intravenous concentration [15].

### 4.5 Pharmacokinetics of early postoperative intraperitoneal chemotherapy (EPIC) fluorouracil

**Fig. 5:**
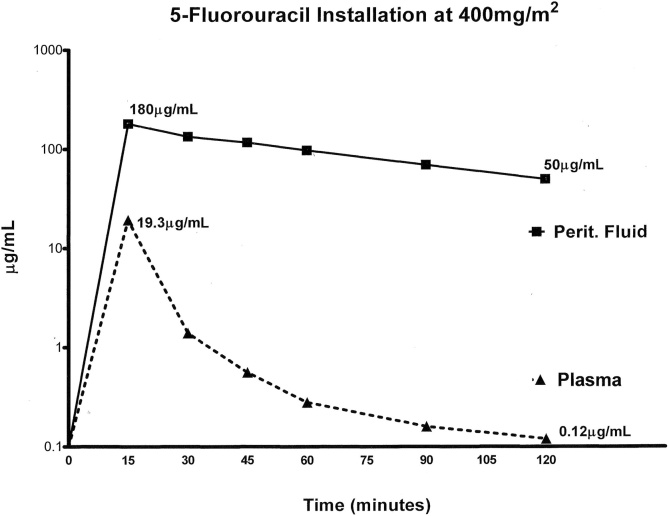
Concentration over time for 5-fluorouracil as early postoperative intraperitoneal chemotherapy (EPIC). After abdominal closure an instillation of 5-fluorouracil at 400 mg/m^2^ in 1 L of 1.5 % dextrose peritoneal dialysis was performed. 5-fluorouracil concentrations in peritoneal fluid and plasma are shown for 2 h. The instillation was continued for 24 h.

After closure of the abdomen and with all transabdominal drains clamped, 5-fluorouracil at 400 mg/m^2^ is instilled as rapidly as possible in one liter of 1.5 % dextrose peritoneal dialysis solution. Fig. 5 shows the pharmacokinetic assessment limited to the first 2 h of the 24 -h dwell of chemotherapy solution within the peritoneal space. During the remainder of the 24 -h dwell, intravenous 5-fluorouracil remained between 0.24 μg/mL to 0.12 μg/mL as a result of continuous infusion and intraperitoneal 5-fluorouracil.

## 5 Discussion

### 5.1 Strategy for management of peritoneal metastases

For colorectal and appendiceal cancer an optimal use of cancer chemotherapy is required to achieve the longest survival of patients with this disease. The chemotherapy can be used prior to a cancer resection in an attempt to downstage the disease. It can be used after the cancer resection as an adjuvant treatment in an attempt to eradicate minimal residual disease. In patients in whom cancer resection is not possible it can be used palliatively to reduce symptoms and prolong life. A new strategy described for a subset of patients who have peritoneal metastases from colorectal or appendiceal cancer is perioperative chemotherapy [11]. In these patients the peritoneal metastases are resected using peritonectomy procedures and visceral resections to make the abdomen and pelvis visibly free of disease [10]. After the surgery has been completed the abdomen and pelvis are flooded by a chemotherapy solution. Also, intravenous drugs may be indicated to maximize this perioperative chemotherapy treatments. The regional dose intensive chemotherapy is used in an attempt to eradicate minimal residual disease that cancer resection cannot eliminate. It is an attempt to use regional chemotherapy to preserve the complete visible response achieved by the cytoreductive surgery.

### 5.2 Requirement for 5-fluorouracil to develop the cytotoxicity of oxaliplatin in perioperative FOLFOX

Oxaliplatin as a single agent has a poor response rate with previously untreated metastatic colorectal cancer. Becouarn et al. reported partial response in approximately 20 % of patients [16]. This is approximately the same as that reported for mitomycin C, doxorubicin or 5-fluorouracil. A revolution in chemotherapy for colorectal and appendiceal cancer was the simultaneous use of 5-fluorouracil combined with oxaliplatin. The incidence of a response of previously untreated colorectal cancer to single agent 5-fluorouracil is estimated at 20 %. Likewise, the incidence of a response to single agent oxaliplatin chemotherapy is approximately 20 %. When these two drugs are combined as a FOLFOX 7 treatment regimen the response rate on previously untreated colorectal cancer patients is approximately 60 % with a durable response up to 3 years [17]. In the FOLFOX 6 and FOLFOX 7 regimen the dose of 5-fluorouracil in 2400 mg/m^2^ by continuous infusion over 48 h [18].

In the Perioperative FOLFOX regimen, the dose of 5-fluorouracil approaches the dose as in the most effective systemic FOLFOX regimens but is for approximately 24 rather than 48 h. At the initiation of the HIPEC oxaliplatin, the bolus of 400 mg/m^2^ 5-fluorouracil is followed by a continuous infusion of 5-fluorouracil at 800 mg/m^2^ over 90 min. At the completion of the cytoreduction after the abdomen is closed, 1 liter of chemotherapy solution containing 400 mg of 5-fluorouracil is instilled into the peritoneal space as the first EPIC treatment. The intraperitoneal oxaliplatin dose is 200 mg/m^2^ for perioperative FOLFOX as compared to 130 mg/m^2^ for FOLFOX.

In the CHIP regimen [7] and the HIPEC oxaliplatin published by Stewart et al. [19], the use of 5-fluorouracil is inadequate or absent. The CHIP regimen uses only 400 mg/m^2^ bolus of 5-fluorouracil and the Wake Forest regimen used no 5-fluorouracil. Only low percentage of response can be expected with intraperitoneal oxaliplatin in the absence of 5-fluorouracil.

### 5.3 Recommended doses of intraperitoneal oxaliplatin as HIPEC

The doses of chemotherapy agents in this pharmacologic quality improvement project have been previously established safe for use in humans. Stewart performed a dose escalation of intraperitoneal oxaliplatin for 120 min of moderate heat. Temperature within the chemotherapy lavage was 41 °C. The dose selected for routine use was 200 mg/m^2^ [19]. Our pharmacologic studies showed that approximately 90 % of the chemotherapy cleared into the body compartment after a 120-minute peritoneal lavage. With this dose escalation study both local toxicities and systemic toxicities were acceptable.

This dosimetry of intraperitoneal oxaliplatin can be contrasted to the drug administration in the PRODIGE 7 protocol. The ultra-high dose of oxaliplatin (460 mg/m^2^) was only about one-third absorbed in the short 30-minute intraperitoneal treatment [20]. The regional dose intensity was markedly reduced as compared to the study by Stewart et al. The area under the curve for these two methods for oxaliplatin administration are different. The PRODIGE 7 administration had a concentration times time (area under the curve) of 5000 as compared to the longer lavage of Stewart et al. with an AUC of 7000.

Although both the Stewart et al. and French protocols had acceptable systemic toxicities, the local-regional toxicities were much different. In the PRODIGE 7 protocol [7] and in a prior report [21] postoperative bleeding at a rate of approximately 10 % was recorded. Despite this toxicity which required return to the operating room, the CHIP protocol was continued.

### 5.4 Perioperative FOLFOX less effective after neoadjuvant FOLFOX shown by *in-vitro* studies

The PRODIGE 7 randomized controlled trial was interpreted to show that CHIP after complete cytoreduction for colorectal peritoneal metastases was ineffective [7]. Perioperative FOLFOX is pharmacologically much different than CHIP, nevertheless it is not recommended to be used after neoadjuvant systemic chemotherapy with FOLFOX. After the systemic chemotherapy has eliminated responsive cancer cells, the tumor that remains should be considered resistant to oxaliplatin plus 5-fluorouracil. These resistant cells after many cycles of neoadjuvant systemic chemotherapy are unlikely to respond to perioperative treatment with identical chemotherapy agents. In our 2 patients, no neoadjuvant systemic chemotherapy had been used. A solid recommendation for patient selection for perioperative FOLFOX is that patients with neoadjuvant FOLFOX are poor candidates for this chemotherapy regimen.

*In-vitro* assessment of cancer chemotherapy response have never been accepted as predictive of benefit. However, if an *in-vitro* assessment shows resistance to a chemotherapy agent, response to this agent is not expected and its use is not indicated. Yonemura and colleagues tested the chemosensitivity/resistance to cancer chemotherapy in cells taken from patients treated with neoadjuvant FOLFOX [22]. Chemosensitivity was not inhibited to mitomycin C, 5-fluorouracil, docetaxel and cisplatin. However, inhibition of oxaliplatin in colorectal cancer tissues after neoadjuvant FOLFOX was significantly greater that tissues from other neoadjuvant chemotherapy treatments or from untreated cancer cells. These researchers concluded that after neoadjuvant chemotherapy using FOLFOX, mitomycin C, 5-fluorouracil, docetaxel and cisplatin could be recommended for perioperative chemotherapy, but not oxaliplatin [22]. A second *in-vitro* study to assess the cytotoxic responses to oxaliplatin after neoadjuvant FOLFOX was reported by Nagourney and colleagues. They used an apoptosis assay to assess drug resistance to oxaliplatin *in-vitro*. Chemotherapy-naïve patients with colon cancer were significantly more responsive to oxaliplatin than those who had received previous FOLFOX (p < 0.01). The degree of resistance increased when the systemic chemotherapy treatments were 2 or fewer months before testing [23].

### 5.5 Use of hyperthermia to augment oxaliplatin

In the HIPEC procedure to treat peritoneal surfaces for small volume residual disease after cytoreduction, a hyperthermic chemotherapy solution is used. Temperatures within the abdominal-pelvic fluid is maintained at 41.5–42.5 °C for 90 min [11]. The drugs used, mitomycin C, doxorubicin, and cisplatin have animal models to show that heat augments the cytotoxicity of these agents. Oxaliplatin is stable to 46 °C so the drug will maintain its activity with hyperthermia. But there are no animal models of peritoneal metastases to suggest benefit from hyperthermia. A single manuscript using the mouse footpad assay suggests in-vivo heat augmentation of oxaliplatin at 41.5 °C if ultrahigh doses of drug are used. Doses of oxaliplatin used clinically are not augmented by hyperthermia [24].

## Conflicts of interest

Paul H. Sugarbaker and O. Anthony Stuart have no conflicts of interest to declare.

## Funding

Data management and secretarial support provided by Foundation for Applied Research in Gastrointestinal Oncology.

## Ethical approval

Local IRB-approval for this case report was not required:

MedStar Health Institutional Review Board has determined that a case report of less than three (3) patients does not meet the DHHS definition of research (45 CFR 46.102(d)(pre-2018)/45 CFR 46.102(l)(1/19/2017)) or the FDA definition of clinical investigation (21 CFR 46.102(c)) and therefore are not subject to IRB review requirements and do not require IRB approval.

This case report is of 2 patients.

## Consent

Written and signed consent was obtained from the patients.

## Author contribution

Paul H. Sugarbaker: study concept or design, data collection, data analysis or interpretation, writing the paper

O. Anthony Stuart: data collection, data analysis or interpretation, writing the paper

## Registration of research studies

Name of the registry: ResearchRegistry.com

Unique identifying number or registration ID: UIN 5425

Hyperlink to your specific registration (must be publicly accessible and will be checked): https://www.researchregistry.com/browse-the-registry#home/registrationdetails/5e6bc5062c28eb0015f10eb7/

## Guarantor

Paul H. Sugarbaker, MD accepts full responsibility for the work and conduct of the study. He has access to the data and controlled the decision to publish

## Provenance and peer review

Not commissioned, externally peer-reviewed
